# 

**Published:** 1889-11

**Authors:** 


### TABLE OF CONTENTS.

ORIGINAL AND SELECTED.

Hyoscine Hydrobromate as a hypnotic, by E. B. Potter, M. D ..... 401
Colon-Flushing in typhoid fever, by A. P. Buchman, M. D ..... 404
On the use of mercuric bichloride ..... 406
Cocaine in eye, ear and throat practice ..... 409
Warning against contagion from consumption ..... 411
Hypnotism in therapeutics ..... 413
The dread of death ..... 415

ABSTRACTS &c.

Chloralamid; Death from chloroform ..... 416
Acetanilide in epilepsy; Treatment of sciatica with electricity; Transplanting a chicken's cornea ..... 417
Dangerous glycerine; Sul. of cal. in phthisis; Treatment of headaches ..... 418
Rules for administering chloroform; Antiseptic power of salol ..... 419
Infantile Diarrhœa ..... 420
Is antifebrin dangerous; Cascara sagrada and glycerin in constipation ..... 421
Mullein and vinegar in phlegmasia dolens; Rheumatic fever ..... 422
Beetroot in habitual constipation and hemorrhoids; Rheumatism; Hydrate of chloral in chapped nipple ..... 423
Scarlet fever; Retrospective therapeutics, upon various topics ..... 424
Ferrum phos. in pneumonia; Glycerine suppositories for constipation; Stannum in headache; Antiseptic treat. diphtheria; Sea-water spray 425
Strychnine in alcoholism; Gossypium herbaceum; Cocoanut as a tænifuge; Modelling clay in mastitis ..... 426
Bromidia as a hypnotic ..... 427
Nitrite of amyl in epilepsy and purtussis ..... 428
Congress of American Physicians and Surgeons; Tannin treatment of phthisis ..... 429

SCIENTIFIC.

Edison says; Experiments with carbo-dynamite Combined phonograph and photograph ..... 430
Extent and population; Cooking by electricity; 85 miles in 82 minutes; Inprovement of the phonograph ..... 431

NOTES AND FORMULÆ.

Eczema; Powder stains on the skin; For summer diarrhœa; Menthol in pruritus; Laxative powder ..... 532
Ophthalmic migraine; Nasal catarrh; Laxative pills; Excessive menstruation; Ulceration of the ear ..... 433

EDITORIAL

A new departure; France decreasing in population; Post graduate instruction ..... 434
Book notices ..... 435
Book notices ..... 436
Mississippi Valley Med. Asso.; Eucalyptol ..... 437
Improved method of amputation of the penis; Syrup of tar; The question of contagion in cancer; wanted, a physician ..... 438
Special notes ..... 439, 440

